# Raman spectroscopy coupled with principal component analysis to quantitatively analyze four crystallographic phases of explosive CL-20†

**DOI:** 10.1039/c8ra02189a

**Published:** 2018-06-27

**Authors:** Xuan He, Yu Liu, Shiliang Huang, Yi Liu, Xuemei Pu, Tao Xu

**Affiliations:** Institute of Chemical Materials, China Academy of Engineering Physics Mianyang 621900 China xuan.hellen@gmail.com xuan.hellen@caep.cn; College of Chemistry, Sichuan University Chengdu 610064 People's Republic of China

## Abstract

The polymorphic quantitative analysis of explosives is very important for national defense and security inspection. However, conventional analytical methods are inaccurate and time-consuming because of the complexity of the polymorphic explosive samples. In this paper, we established a new method of polymorphic quantitative determination in a simple, sensitive, and accurate way. High quality spectra of the four phases of the explosive CL-20 were obtained using a compact Raman spectrometer, and QM calculations were performed to confirm the tentative assignment of the most predominant Raman peaks. Principal component analysis (PCA) of the data was performed to understand the factors affecting the spectral variation across the entire Raman region of the four phases of CL-20 and to calculate the characteristic Raman shift region. In addition, different characteristic peaks were selected according to the PCA and QM calculation results, and a new method for the quantitative determination of polymorphic impurities in ε-CL-20 was also set up. The detection level for the polymorphic impurities was determined to be below 1%, and the standard deviation was less than ±0.5%. This new method is of significant importance for the quality control of synthesis and production not only in explosives, but also in pharmaceuticals, agrochemicals, and optics industries.

## Abstract 2

Principal component analysis (PCA) of the data was performed to calculate the characteristic Raman shift region of CL-20, and a new method for the quantitative determination of polymorphic impurities in ε-CL-20 was set up.

## Graphical abstract

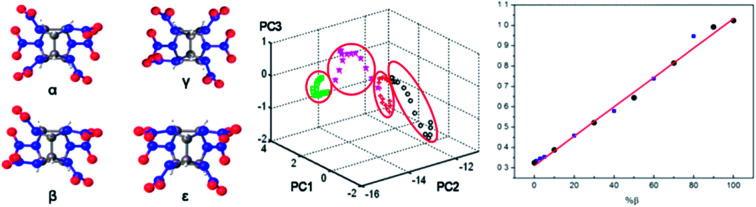

## Introduction

In materials science, because of different molecular arrangements in the crystal lattice, polymorphism is considered as the ability of a compound to crystallize in more than one form.^1^ Different polymorphs of drugs, explosives, agrochemicals, pigments, and dyestuffs can exhibit dissimilar physical and chemical properties, many of which may translate into differences in actual applications.^2–6^ The ability to detect the presence of different polymorphs in a sample, assay their relative amounts, and ultimately obtain the desired form in very high yield is of crucial importance in chemical engineering and technology.^7–10^

Raman scattering is a powerful method to investigate polymorphs due to its remarkable sensitivity to the crystalline structure of materials.^11–15^ The ability to determine the simultaneous presence of multiple polymorphs in the same sample by distinguishing their Raman spectra is a challenge in polymorph characterization because the vibration frequency of Raman peaks is very weak, complex, and often hard to distinguish. Therefore, we need to introduce theoretical calculations for a more accurate spectral analysis.

Principal component analysis (PCA) is a classic multivariate technique that analyses a data table where observations are described by several inter-correlated quantitative dependent variables.^16,17^ PCA of the data is performed to understand the factors affecting the spectral variation across the samples, and it is calculated from the covariance matrix of the original data set. PCA is an axis rotation technique that aligns a new set of axes, called principal components (PCs), with the maximal directions of variance within a dataset. Thus, this method creates three new matrices containing the scores, the loadings, and the residuals.^17,18^ The score plot shows whether the samples or a group of samples are different. The corresponding loading plot shows the difference in the Raman spectra and different polymorphs of the samples. Therefore, using the principal component analysis method, the purpose is to interpret the complex Raman spectra of different polymorph compounds by revealing differences between the samples (expressed as so-called “scores”) and relating them to differences in the variables (called “loadings”) defining a sample.^19–22^ Successful applications of PCA to understand the factors affecting spectral variation have been reported.

**Fig. 1:**
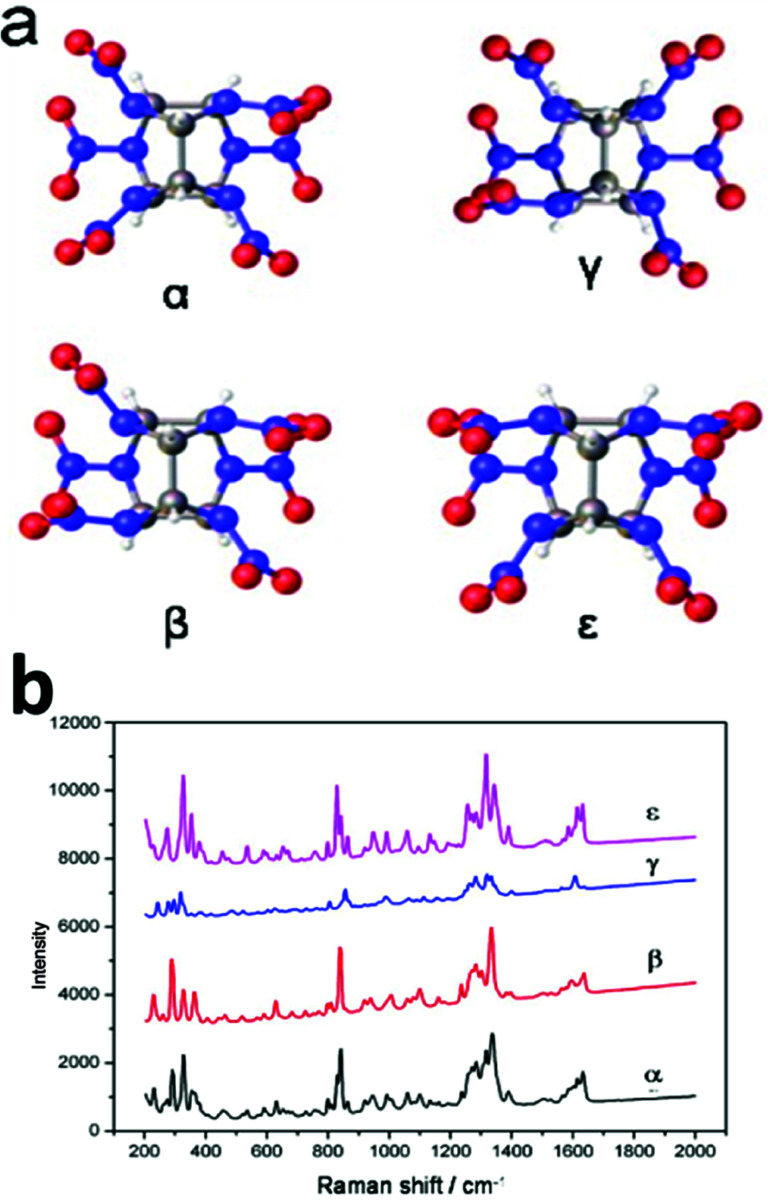
(a) Structure of the caged nitramine 2,4,6,8,10,12-hexanitro-2,4,6,8,10,12-hexaazaisowurtzitane (CL-20). (b) Raman spectra of the four different phases of CL-20 (α, β, γ, and ε).

Fig. 1a shows one of the most recently developed energetic materials: the caged nitramine 2,4,6,8,10,12-hexanitro-2,4,6,8,10,12-hexaazaisowurtzitane (CL-20). There are four different polymorphic phases at ambient pressure and temperature: α, β, γ, and ε. The ε-phase is the most thermodynamically stable and the highest density energetic material.^23–25^ In this study, we characterized the four phases of the CL-20 explosive by Raman spectroscopy using the PCA method to draw the main spectroscopic information. A tentative assignment of the most predominant Raman peaks were recorded with the aid of QM calculations at the B3LYP/6-311G(d) level. Different characteristic peaks were selected according the PCA and QM calculation results, and a new method for the quantitative determination of polymorphic impurities in ε-CL-20 was also developed. The detection level for polymorphic impurities was determined to be below 1%. This new method is of significant importance for the quality control of synthesis and production not only in explosives, but also in pharmaceuticals, agrochemicals, pigments, dyestuffs, food, energy storage, biominerals, and nonlinear and optics industries.

## Experimental

### Apparatus and software

The crystal structure of the products was studied using a Bruker D8 X-ray diffractometer with Cu Kα irradiation at *λ* = 1.5406 Å. The Raman test conditions were the following: laser wavelength, 532 nm; laser energy, 5 mW; wavenumber range, 3500–50 cm^−1^; resolution, 32-times the 4 cm^−1^-background acquisition; number of samples, 32. DXR Smart Raman Nicolet spectrometer was used for the measurements. To check the sample homogeneity, all analyses of the polymorph mixtures were performed in triplicate. The spectra shown are averages of the triplicate runs.

All data obtained from the experiments were gathered in a data matrix using Microsoft Office Excel (version 2010) and transferred to the MATLAB software. All calculations were conducted using MATLAB (version 2013 a).

### Sample preparation

#### PCA calculations

CL-20 samples (3 mg) were packed into a sample cup for analysis. The Raman spectrogram of the α, β, γ, and ε-phase of CL-20 were utilized to construct the calibration set (Fig. 1b). The calibration set consisted of 60 samples, with 15 samples of each phase.

#### Analysis of polymorphic purity

In brief, 0.1 g of pure β- and ε-polymorphs of CL-20 were weighed, mixed, and ground.Two groups of samples were prepared: the first group: β-100%, β-10% + ε-, β-20% + ε-, β-30% + ε-, β-40% + ε-, β-50% + ε-, β-60% + ε-, β-70% + ε-, β-80% + ε-, β-90% + ε-, ε-100%, the second group: β-1% + ε-, β-3% + ε-, β-5% + ε-. Another two-phase sample was prepared in the same way.

## Results and discussion

### The calibration set: construction of PCA models

#### Experimental design

The XRD and Raman spectra of the four phases of CL-20 (α, β, γ, and ε) are shown in Fig. S1 in the ESI† and in Fig. 1b. The Raman spectra of all four stable phases of CL-20 were measured from 50 to 3500 cm^−1^. The measured Raman spectra usually contain two types of noise: one is the additive noise from external conditions, and the other is the background noise from auto-fluorescence. These noises should be carefully treated because they have a strong effect on the performance of the analysis system. Prior to PCA, all spectra were smoothed and normalized to an equal average intensity value, which was then subtracted from each spectrum. After the noise reduction, PCA was performed.

The 60 spectra (15 spectra for each phase) were divided into four groups and then, they were added to the algorithm program. By taking advantage of PCA, we can clearly see the most significant differences in the Raman spectra of the four crystal types of CL-20, thus providing strong support for the establishment of the standard method of crystal quantitative analysis. Moreover, the final goal of this study was to develop a novel strategy to identify the different phases of the CL-20 explosive using Raman spectroscopy coupled with PCA. The elemental composition of CL-20 with different phases can be visualized by two- or three-dimensional cluster analysis to statistically reduce the dimensionality of the elemental composition data to a smaller set of theoretically meaningful component variables. Visual classification based on the first three principle components for the four binary classifiers was carried out for the discriminate analysis of the feature vectors obtained from the Raman spectral data.

The training data (Raman spectra from 50 to 3500 cm^−1^) was included in the corresponding PCA except for the spectra from an individual cell dish. Those Raman spectra usually contain a large number of signals that add complexity to the classification problem. Therefore, the set of spectra was arranged in such a way that each row contained the mean-centered spectrum of a single sample, and the set was polished to remove the less useful regions. A 120 × 514 (row × column) matrix was established. Subsequently, we obtained a score plot analysis of the principal components (PCs) of the input matrix. As shown in the calculation process, the input matrix takes into consideration up to six PCs, as the first PC describes only 51.01% of the data variation, confirming the complexity of the system.

**Fig. 2:**
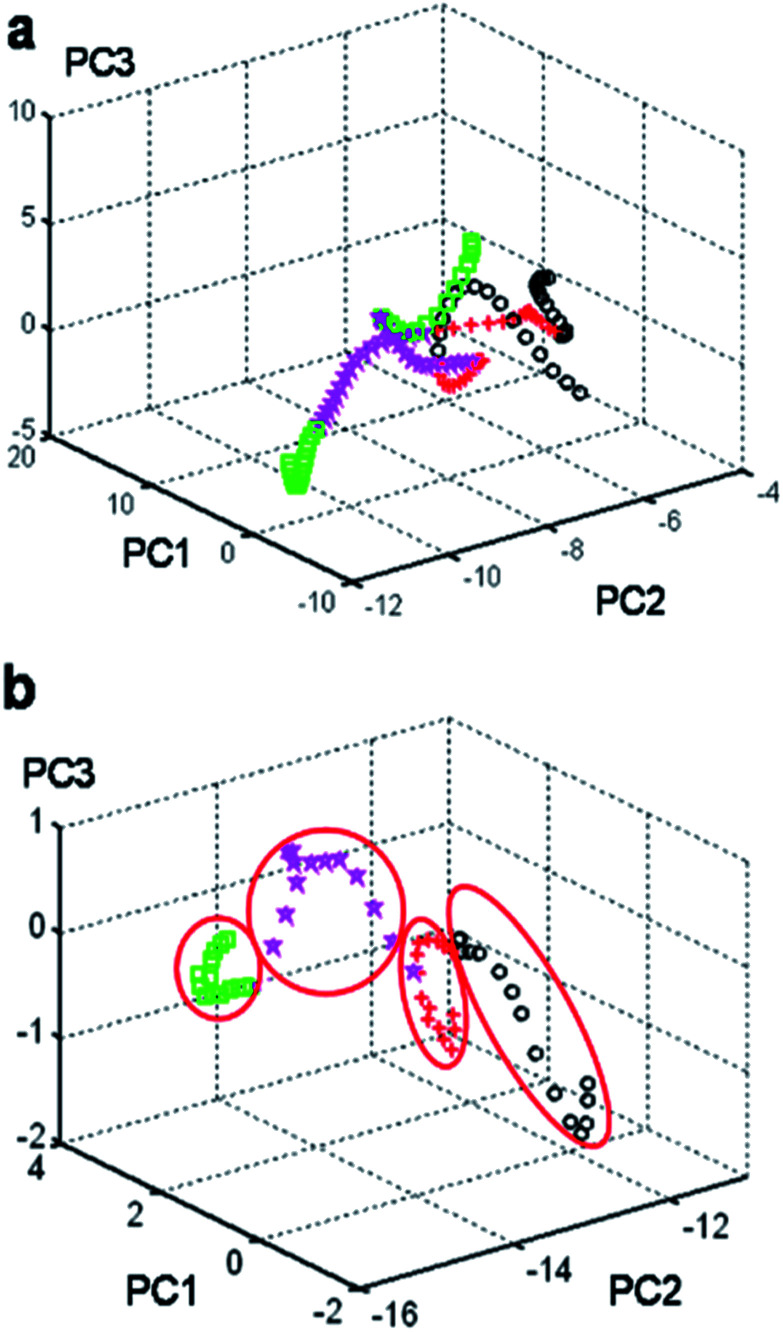
(a) MAP classification showing that the input feature vectors are mixed in the group region from 50 to 3500 cm^−1^ for the four phases of CL-20. (b) MAP classification showing that the input feature vectors are well separated in the group region from 800 to 1000 cm^−1^ for the four phases of CL-20. (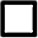: α-CL-20; 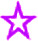: β-CL-20; 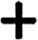: γ-CL-20; 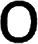: ε-CL-20).

Next, examination of the cumulative variance plot revealed that three components reflected 89.79% of the system variation and six components accounted for 91% of the variance of the system. In Fig. 2, the MAP classification shows that the separation between the different phases of CL-20 was not very clear. According to these calculation results, we should classify the spectral regions and select the specific regions by the PCA method to obtain the 3D feature classification map of the region.

### Selection of the number of factors and optimized calculated models

**Fig. 3:**
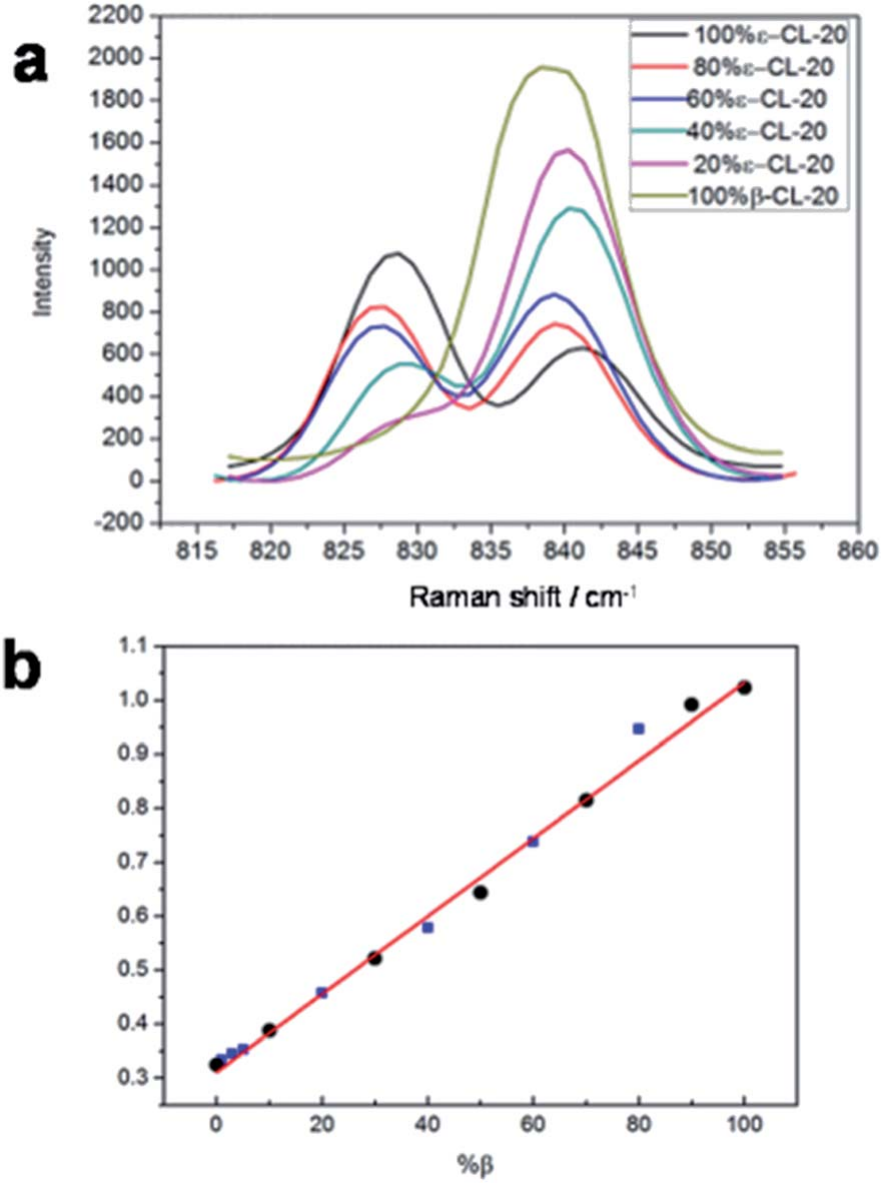
(a) Raman spectra of CL-20 (mixtures of β and ε) in the 810–870 cm^−1^ spectral region; (b) linear relationship between the Raman intensity and the logarithm of the mixtures of β- and ε-CL-20.

Next, a tentative assignment of the peaks was completed with the assistance of quantum mechanics (QM) calculations, and the structure was optimized at the B3LYP-6-311G++(d,p) level of theory using Gaussian 03 (see Fig. 1a and Table S1†). Interestingly, the peaks were unique to the different polymorphs. In the full spectra (50 to 3500 cm^−1^), there were several characteristic peaks for the various polymorphs. It was evident that it is possible to determine the polymorphic identity of an unknown sample of CL-20 by detailed examination. It is worth mentioning that there are strong peaks for all four isomers, probably ascribed to the ring stretching in the cage and to NO_2_ scissoring in the area around 800 cm^−1^ (Fig. 3a).^26^ There are characteristic peaks for three of the four isomers in this area. The bands at 848 cm^−1^, 838 cm^−1^, and 828 cm^−1^ correspond to the characteristic bands of α-, β-, and ε-phases, respectively. Another area with strong bands ranges from 260 to 300 cm^−1^, in which in the area from 280 to 290 cm^−1^, there are peaks corresponding to all polymorphs except for the ε-phase.^27^ As a result, the 800–850 cm^−1^ and 250–300 cm^−1^ areas are the key regions that require further the analysis.

Next, a series of 60 independent samples with Raman spectra from 50 to 3500 cm^−1^ was split into 7 groups (50–200 cm^−1^, 200–400 cm^−1^, 400–800 cm^−1^, 800–1000 cm^−1^, 1000–1500 cm^−1^, 1500–2500 cm^−1^, and 2500–3500 cm^−1^). After calculations and based on the MAP classification results, the input matrix indicated that up to six PCs should be taken into consideration as the first PC described only 76.05% of the data variation. Moreover, examination of the cumulative variance plot revealed that three components reflected 97.07% of the system variation, but six components accounted for 99.86% of the variance of the system. The MAP classification clearly showed that the input feature vectors were mixed and could not be separated in the group region from 50–3500 cm^−1^ (Fig. 2a). However, they could be well separated using this feature space in the group region from 800–1000 cm^−1^ (Fig. 2b). The other calculated results are shown in the ESI† Fig. S3–S8.† The position of each phase of the CL-20 compound was reported in the three-dimensional space for the three principal components (PC1, PC2, and PC3). Consequently, the area from 800–1000 cm^−1^ was the feature region that displayed the maximal difference. Moreover, the quantum mechanics (QM) calculation (tentative assignment of peaks) results showed strong peaks in all four isomers, probably relating to the ring stretching in the cage in the area around 800 ± 100 cm^−1^ (Table S1†). Analysis of the Raman spectra of all four polymorphs prompted us to develop a new method for the polymorphic purity analysis of ε-CL-20.

### New method for polymorphic detection

The linear relationship between the ε-CL-20 content and that of the other phases of CL-20 was established based on the relative peak height of the ring stretching in the cage and the NO_2_ scissoring. The following equation is obtained:*X* = *a* + *kY*where *X* is the mixture content of α-, β- or γ- in ε-CL-20, a is a constant, *k* is the coefficient, and *Y* is the peak of the two strong rates of change, which is determined as*Y* = *I*_ε_ / *I*_ε_ + *I*_C_where *I* is the Raman intensity of the characteristic peak of each phase of CL-20, C is α-, β- or γ-CL-20. Furthermore, the 810–870 cm^−1^ region of the Raman spectra was analyzed. The overlay of the 810–870 cm^−1^ expansion of the spectra generated is displayed in Fig. 3a. A series of experiments were performed on mixtures of β- and ε-CL-20, which were varied from 100% β- to 100% ε- (β-100%, β-10% + ε-, β-20% + ε-, β-30% + ε-, β-40% + ε-, β-50% + ε-, β-60% + ε-, β-70% + ε-, β-80% + ε-, β-90% + ε-, and ε-100%). Fig. 3b illustrates the linear relationship between the Raman intensity and the logarithm of the mixtures of β- and ε-CL-20 in the range of β-100%, β-10% + ε-, β-30% + ε-, β-50% + ε-, β-70% + ε-, β-90% + ε-, and ε-100%. The peak of the two strong rates of change, *Y*, was *I*_838_/*I*_838_ + *I*_828_, where *I*_828_ is the intensity of the peak at 828 cm^−1^ of ε-CL-20 and *I*_838_ is the intensity of the peak at 838 cm^−1^ of β-CL-20. The overlay of the 800 ± 100 cm^−1^ expansion of the generated spectra is displayed in Fig. 3b. From the linear regression equation *y* = 7.442 − 0.738*x*, the square of the correlation coefficient was calculated to be 0.9974. The standard deviation was 0.0158, *N* = 3. Then, more samples of the mixtures of β/ε in β-20% + ε-, β-40% + ε-, β-60% + ε-, and β-80% + ε- were detected and calculated into the linear regression equation. The standard deviation was less than 3%.

### Applications to analysis of polymorphic purity

**Fig. 4:**
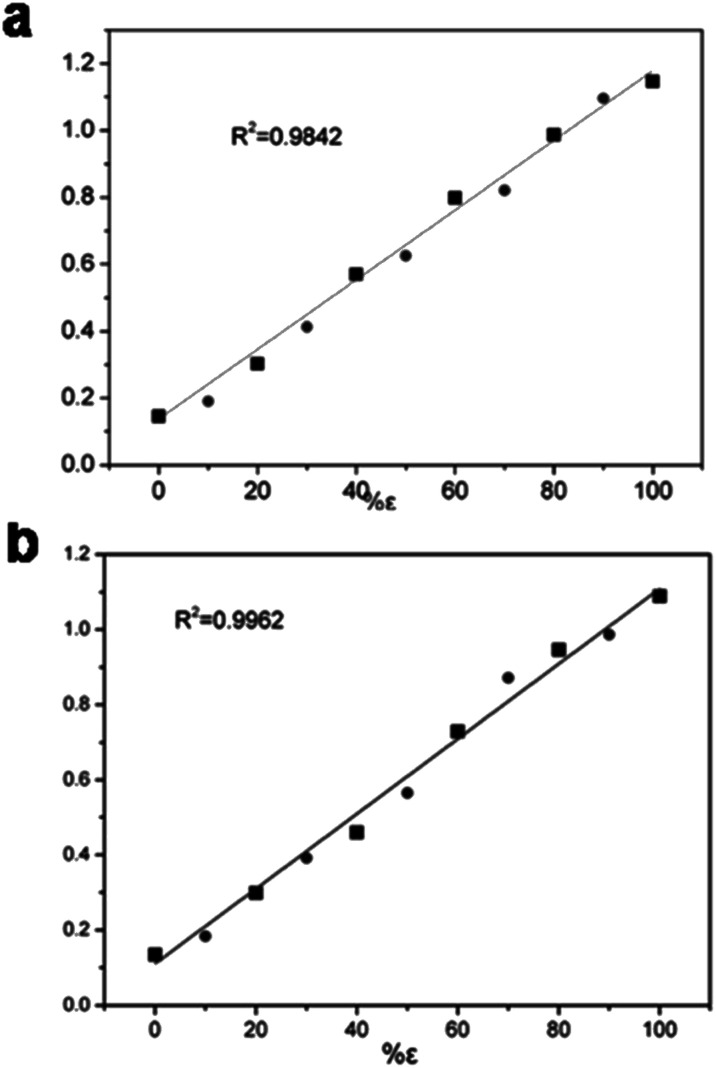
(a) Linear relationship between the Raman intensity and the logarithm (*I*_838_/*I*_838_ + *I*_828_) of the mixtures of α- and ε-CL-20. (b) Linear relationship between the Raman intensity and the logarithm (*I*_838_/*I*_838_ + *I*_828_) of the mixtures of γ- and ε-CL-20.

It is of significance to check the polymorphic purity of the ε-phase for very small amounts of the mixture phase.^28,29^ The real samples were made by varying the amount of β-phase in the ε-phase from 1, 3, to 5%. It was possible to manually detect 1% of the β-phase in the ε-phase, and the standard deviation was less than ±0.5% (Fig. 3b). To further ascertain that this quantitative analysis method had good applicability, we prepared other two-phase mixtures (α and γ-phase) in ε-phase CL-20 with a similar method that is detailed in the ESI† Fig. S9 and S10.† The linear relationship between the Raman intensity and the logarithm of the mixtures of α- and ε-CL-20 and the mixtures γ- and ε-CL-20 are shown in Fig. 4, Table S3 and S4;† the standard deviation was less than ±2.0% and ±2.5%, respectively. Therefore, Raman spectroscopy and PCA calculation methods could be used for the quantitative analysis of low-content crystalline samples in ε-CL-20. The results suggested that this methodology is a promising and useful alternative for classifying and assigning the polymorphic identity of other explosives in a complex system.

## Conclusions

In summary, a simple, sensitive, and accurate method for the polymorphic quantitative analysis of four different phases of the explosive CL-20 was developed. A tentative assignment of the most predominant Raman peaks was made with the aid of QM calculations at the B3LYP/6-311G(d) level. The different characteristic peaks were selected based on the PCA analysis and QM calculation results. A new method for the quantitative determination of polymorphic impurities in ε-CL-20 was also established. The detection level for polymorphic impurities was determined to be below 1%, and the standard deviation was less than 0.5%. Other two-phase CL-20 mixtures in the ε-phase were also detected. This new method is of significance for quality control of synthesis and production not only in explosives, but also in pharmaceuticals, agrochemicals, pigments, dyestuffs, food, energy storage, bio-minerals, and nonlinear and optics industries.

## Conflicts of interest

There are no conflicts to declare.

## Supplementary Material

RA-008-C8RA02189A-s001
