# Supplementary material for: Raman spectroscopy coupled with principal component analysis to quantitatively analyze four crystallographic phases of explosive CL-20
Source: RSC Adv. 2018 Jun 27;8(41):23348–52. doi: 10.1039/c8ra02189a (PMC9081593; doi:10.1039/c8ra02189a)
Supplement: RA-008-C8RA02189A-s001 [file RA-008-C8RA02189A-s001.pdf]

Electronic Supplementary Material (ESI) for RSC Advances.
This journal is © The Royal Society of Chemistry 2018

Supporting Information

XRD experiments

Figure S1. XRD patterns of CL-20.

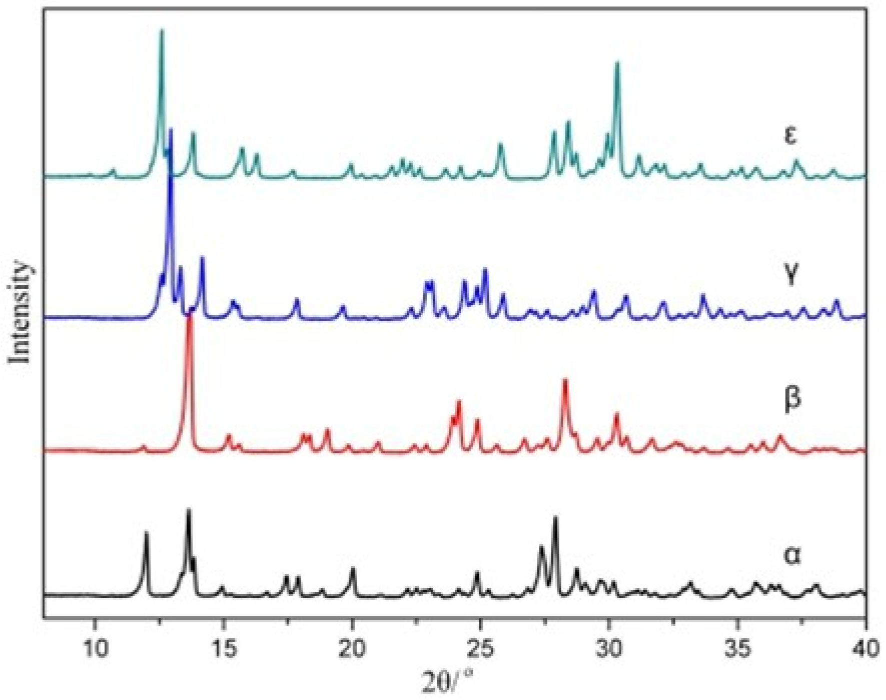

Figure S2. Raman spectra of CL-20.

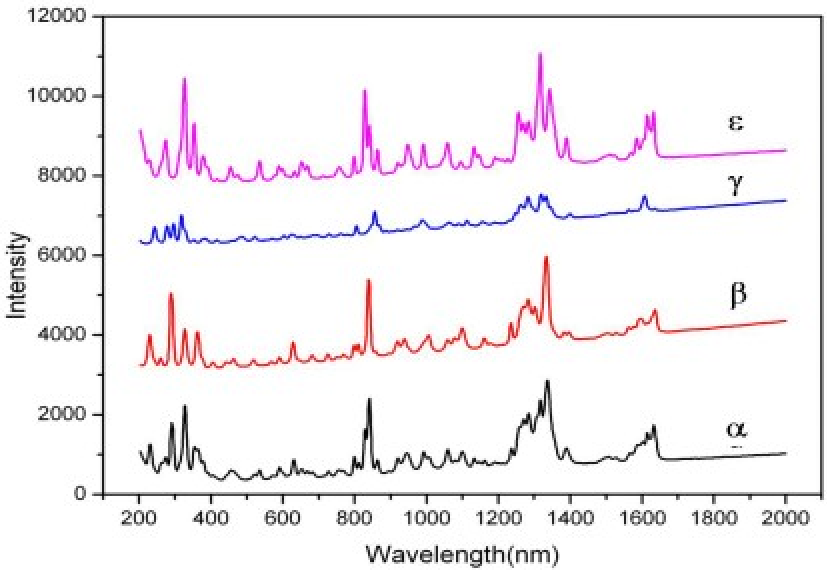

Figure S3. Three-dimensional scattered plot for the PCA-transformed Raman spectra of 50-200 cm^{-1} region for four phase CL-20.

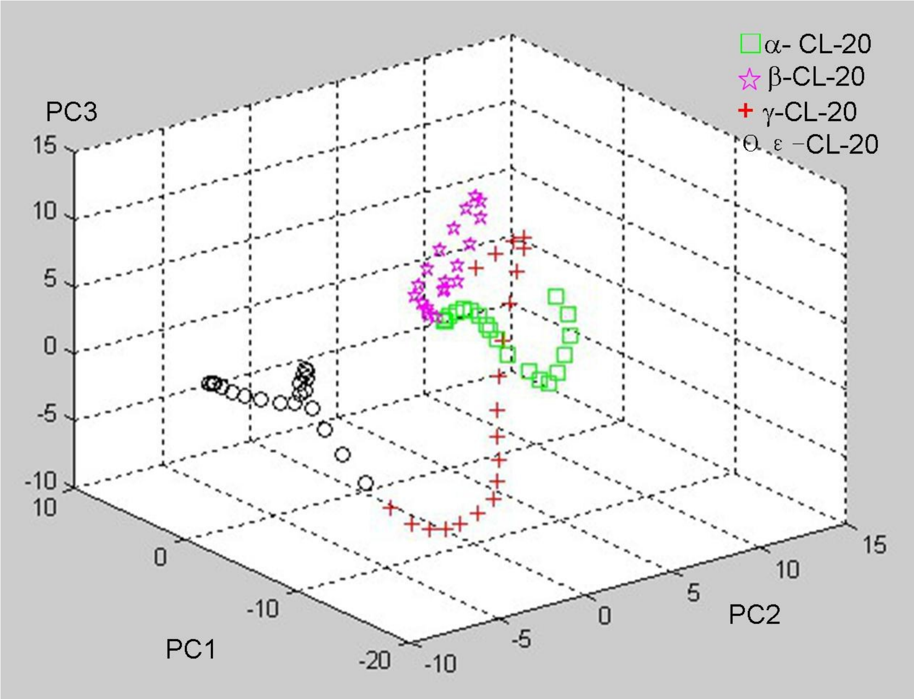

Figure S4. Three-dimensional scattered plot for the PCA-transformed Raman spectra of 200-400 cm^{-1} region for four phase CL-20.

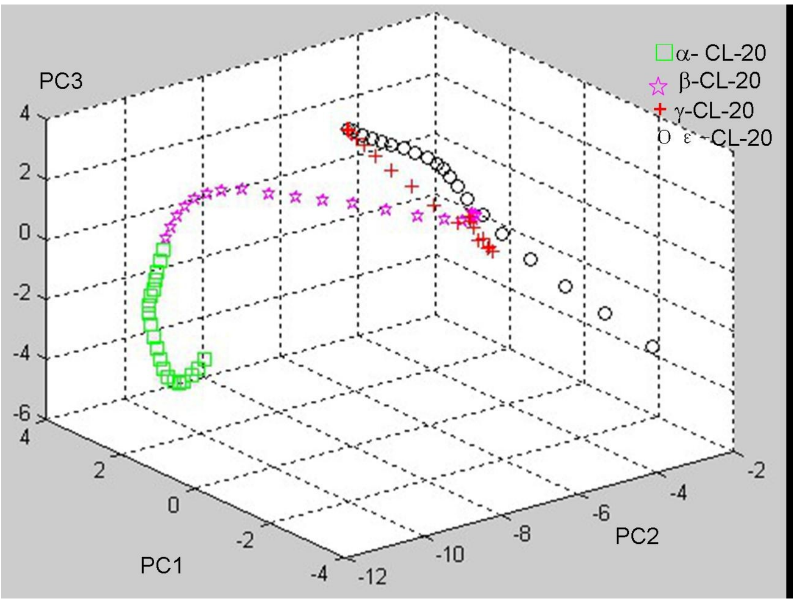

Figure S5. Three-dimensional scattered plot for the PCA-transformed Raman spectra of 400-800 cm^{-1} region for four phase CL-20.

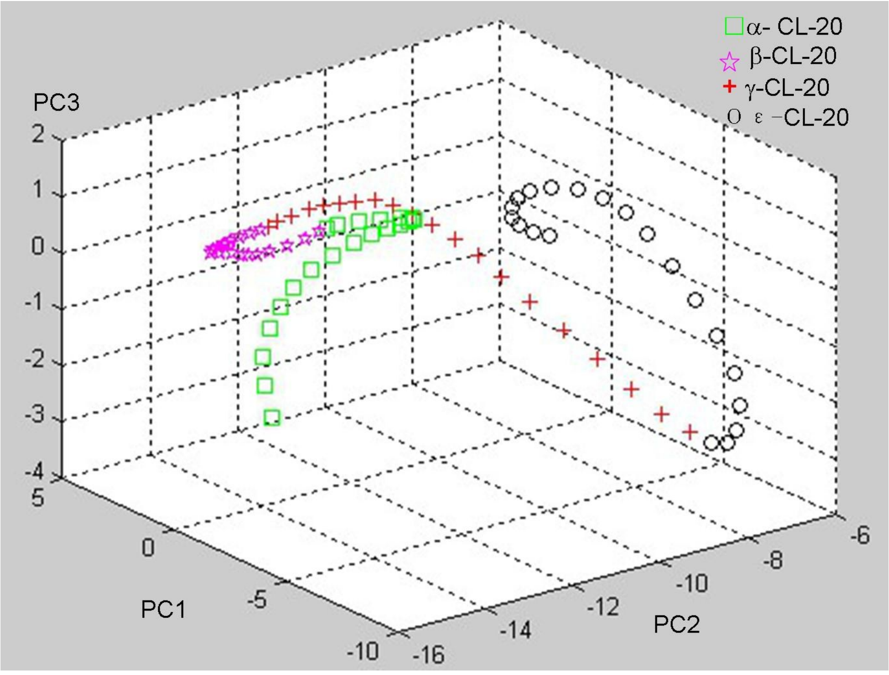

Figure S6. Three-dimensional scattered plot for the PCA-transformed Raman spectra of 1000-1500 cm^{-1} region for four phase CL-20.

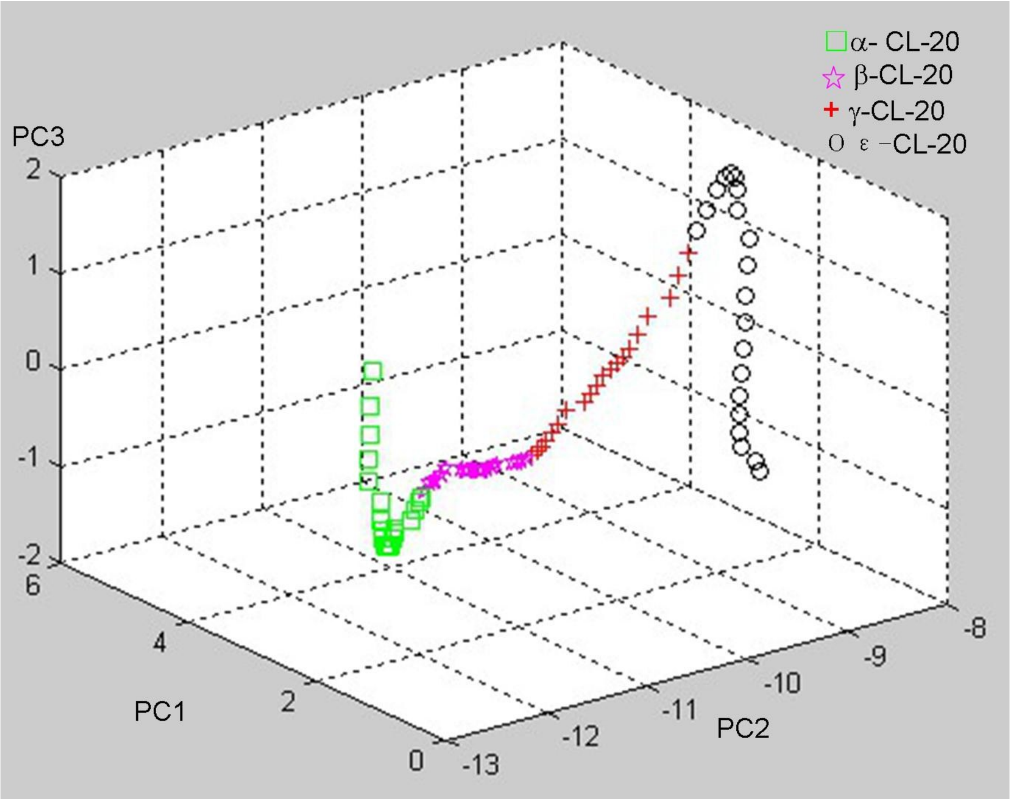

Figure S7. Three-dimensional scattered plot for the PCA-transformed Raman spectra of 1500-2500 cm^{-1} region for four phase CL-20.

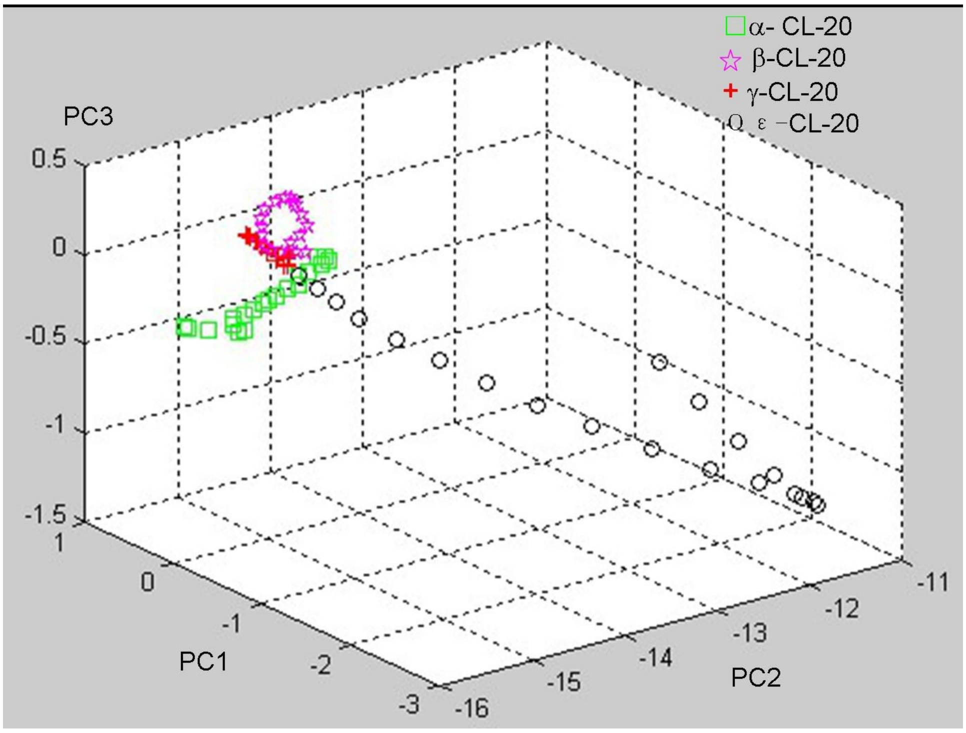

Figure S8. Three-dimensional scattered plot for the PCA-transformed Raman spectra of 2500-3500 cm^{-1} region for four phase CL-20.

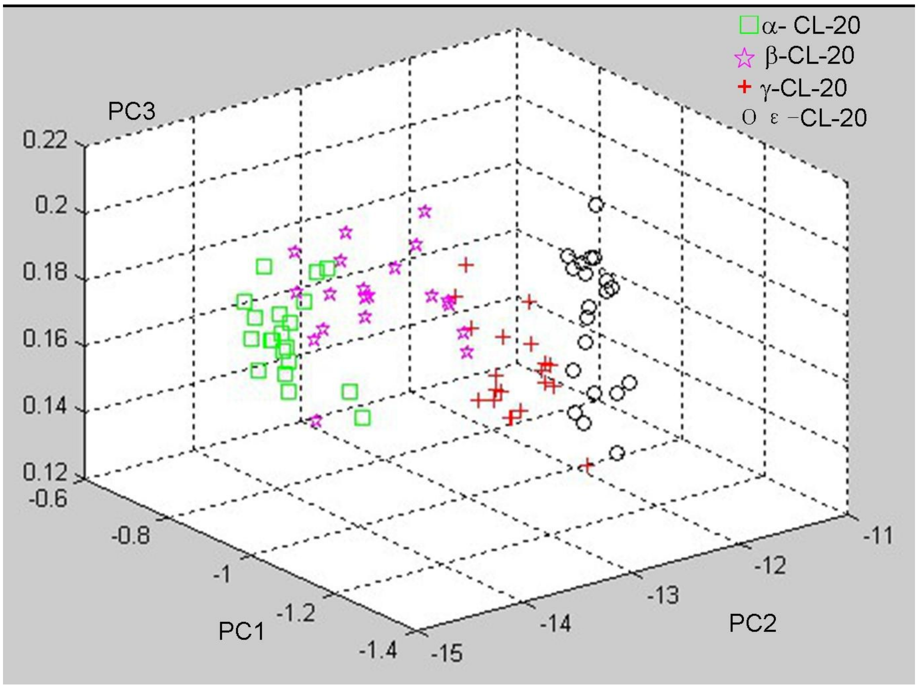

Figure S9. Raman spectra for CL-20 (mixtures of γ and ε) in the 50-2000 cm^{-1} spectral region.

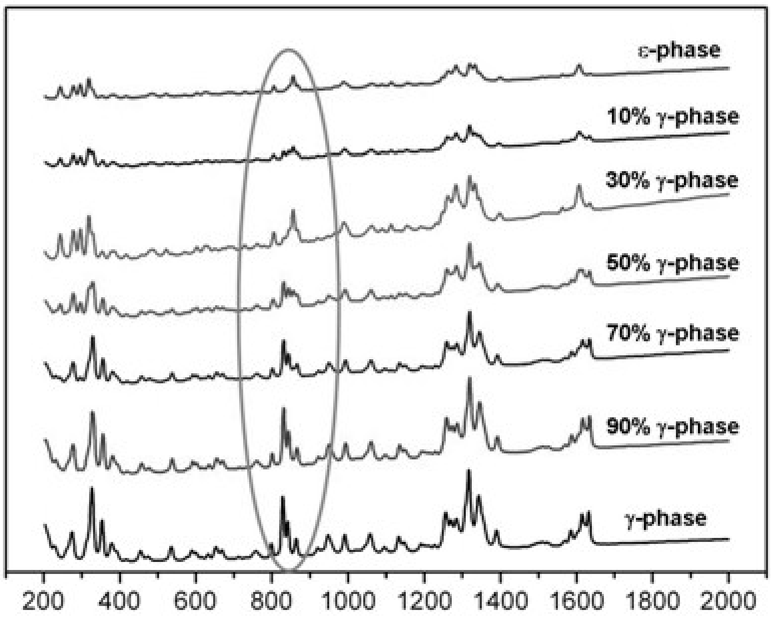

Figure S10. Raman spectra for CL-20 (mixtures of α and ε) in the 50-2000 cm^{-1} spectral region.

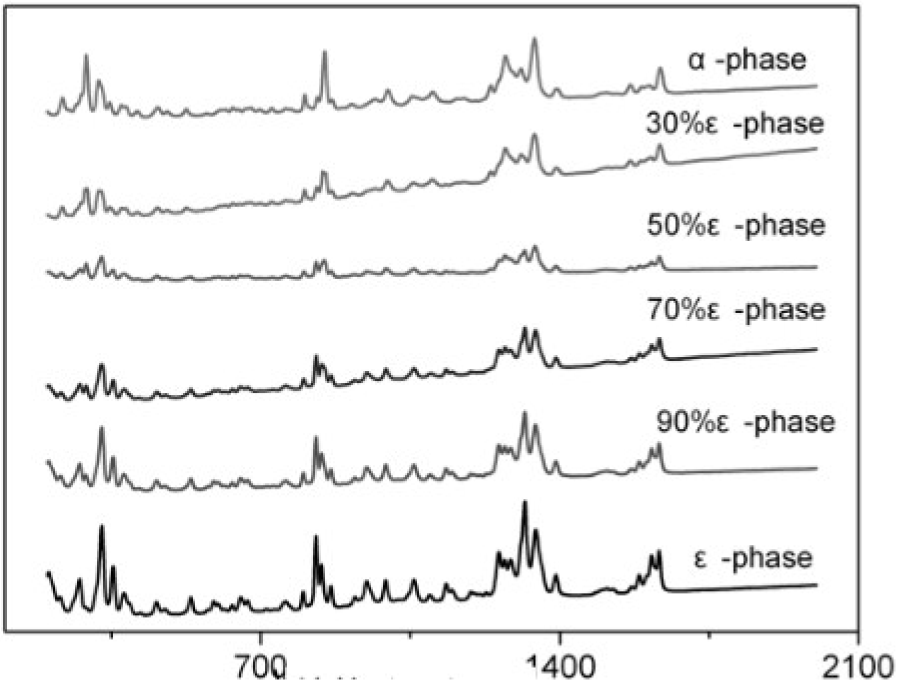

Table S1 Assignments of Raman peaks for CL-20[1]

Raman Shift/cm^{-1}	Vibrational Mode
250-350	CL-20 "Cage" stretch
810-870	Ring stretch
	NO2 scissoring
1050	N-N stretch
1200-1400	Symmetric NO2 stretch
1550-1680	Anti-symmetric NO2 stretch
3010-3070	C-H stretch

Table S2 the detection sample and deviation

Sample	Sample quality	The actual proportion %β	The calculation proportion %β	Deviation(+-)
A	5mg	1	0.8	0.2%
B	5mg	3	3.4	0.4%
C	5mg	5	4.7	0.3%
D	5mg	20	19.8	0.2%
E	5mg	40	38.8	1.2%
F	5mg	60	59.5	0.5%
G	5mg	80	83.7	2.7%

Table S3 the detection sample and deviation

Sample	Sample quality	The actual proportion % ε	The calculation proportion % ε	Deviation(+-)
A	5mg	10	8.2	1.8%
B	5mg	30	28.9	1.1%
C	5mg	50	48.8	1.2%
D	5mg	70	69.0	1.0%
E	5mg	90	90.8	0.8%
A	5mg	10	8.2	1.8%
B	5mg	30	28.9	1.1%

Table S4 the detection sample and deviation

Sample	Sample quality	The actual proportion % ε	The calculation proportion % ε	Deviation(+-)
A	5mg	10	8.4	1.6%
B	5mg	30	29.7	0.3%
C	5mg	50	48.3	1.7%
D	5mg	70	72.5	2.5%
E	5mg	90	89.2	0.8%
A	5mg	10	8.4	1.6%
B	5mg	30	29.7	0.3%

Reference

[1] P. Goede, N. V. Latypov, H. Östmark, Propellants, Explosives, Pyrotechnics 2004, **29**, 205-208.
